# Long-term persistence, safety and effectiveness of nusinersen in spinal muscular atrophy: a population-based study

**DOI:** 10.1007/s00415-026-13965-0

**Published:** 2026-06-29

**Authors:** Karolina Aragon-Gawinska, Nancy Carolina Nungo-Garzon, Nuria Muelas, Rafael Sivera, Teresa Sevilla, David Hervas, Inmaculada Pitarch-Castellano, Juan F. Vazquez-Costa

**Affiliations:** 1 Neuromuscular Research Unit, Instituto de Investigacion Sanitaria la Fe, Valencia, Spain; 2 Neuromuscular Unit, Department of Neurology, Hospital Universitario y Politecnico La Fe, Avenida Fernando Abril Martorell 106, 46026 Valencia, Spain; 3 Department of Medicine, University of Valencia, Valencia, Spain; 4 Centro de Investigacion Biomedica en Red de Enfermedades Raras (CIBERER), Valencia, Spain; 5 Universidad Carlos Herrera, Moncada, Spain; 6 Department of Applied Statistics and Operations Research, and Quality, Universitat Politecnica de Valencia, Valencia, Spain; 7 Department of Pediatric Neurology, Hospital Universitario y Politecnico La Fe, Valencia, Spain

**Keywords:** Spinal muscular atrophy, Nusinersen, Population-based study, Persistence, SMA-FCR

## Abstract

**Background and objective:**

Nusinersen was the first disease-modifying treatment approved for spinal muscular atrophy (SMA). However, long-term results of broad unselected populations—particularly adolescents and adults—remain limited. We aimed to evaluate nusinersen long-term persistence and effectiveness in a population-based cohort.

**Methods:**

We conducted a population-based, ambispective observational study of all SMA patients in the Valencian community (Spain) between 2017 and 2022, with follow-up until December 2025 or censoring (due to death, clinical trial, or treatment switch). Demographic, clinical, and motor outcomes using revised SMA Functional Composite Score (SMA-FCR) were collected. Patients were classified as responders or non-responders. Nusinersen discontinuation risks and motor trajectories were evaluated using Bayesian linear and mixed linear models.

**Results:**

Of 72 patients included, 18 were < 12 years old (all treated with nusinersen) and 54 were ≥ 12 years (28 treated; 26 untreated) at the baseline visit. After a median follow-up of 4.6 years until censoring, all children were found responders, compared with 68% of those ≥ 12 years. Discontinuation rates were 11% in children compared to 75% in the older cohort. In patients ≥ 12 years, reasons for discontinuation included: treatment burden (71%), and loss (53%) or lack of benefit (43%). Lower baseline SMA-FCR (expEstimate = 0.84 [0.718,0.93],prob:1) and older age (expEstimate = 1.028 [1.011,1.055],prob:1) independently predicted higher discontinuation risk. Sustained treatment was associated with SMA-FCR increase, while untreated and discontinued patients showed slight deterioration.

**Discussion:**

Nusinersen persistence was high in children but declined significantly after age 12 due to treatment burden and limited efficacy although a 25% of adolescents and younger adults with higher baseline function experienced sustained benefit.

**Supplementary Information:**

The online version contains supplementary material available at 10.1007/s00415-026-13965-0.

## Introduction

Spinal muscular atrophy (SMA) is an inherited autosomal-recessive disorder caused by biallelic mutations in *SMN1* gene [1], leading to motoneurons loss, which ultimately results in a progressive weakness and atrophy of skeletal muscles. The paralogous *SMN2* gene is one of the strongest disease modifiers as the number of *SMN2* copies correlates directly with disease phenotype [2]. Classically, four SMA types have been distinguished: SMA type 1 manifesting in the first 6 months of life with rapid motor decline, respiratory insufficiency and high infant mortality in absence of treatment [3]; SMA type 2, appearing between 6 and 18 months of life with patients never achieving independent walking and presenting severe motor disability and scoliosis; SMA type 3, where symptoms occur after patients have acquired the ability to walk; and SMA type 4, where symptoms start in the third decade of life [4]. Patients with 2 *SMN2* copies typically develop severe type 1, patients with 3 *SMN2* copies mostly present with SMA type 2 and patients with 4 or more *SMN2* copies usually present as SMA type 3, or less likely, type 4 [2].

However, the traditional classification based on age at onset and highest motor milestone achieved has important limitations because of existence of intermediate forms, evolving phenotypes during natural course of the disease and even more with the appearance of disease-modifying therapies. For these reasons, another functional classification of patients as walkers, sitters, and non-sitters is preferred [5] as it captures patient’s real-time abilities and management needs. In its natural history, SMA is characterized by motor decline; patients almost never naturally gain major milestones after symptom onset and frequently lose existing abilities over time, effectively falling from higher functional categories to lower ones (e.g., from walker to sitter) [3]. Crucially, the introduction of DMTs has fundamentally altered this paradigm by enabling patients to achieve unprecedented motor milestones and shift upward to higher functional categories [6].

The approval of nusinersen, an antisense oligonucleotide (ASO) and the first regulatory-approved drug for SMA, has greatly impacted patients’ prognosis and changed the SMA landscape forever. Clinical trial data and their open-label extensions *(NCT 02594124)* showed a remarkable benefit in survival, motor milestones, and motor scales for infants and children up to 12 years old treated with nusinersen [7–9], with only mild adverse events. Subgroup analysis in those studies showed greater benefit in patients receiving treatment earlier. Consequently, a study conducted in presymptomatic patients showed outstanding results with patients with 2 copies acquiring motor milestones never observed in natural history and patients with 3 copies having a nearly normal motor development [10]. Despite this evidence, no randomized trials of nusinersen in older populations have been conducted to date.

Initial real-world studies began to fill critical gaps regarding nusinersen’s safety, tolerability, and effectiveness in adolescents, adults, and more severely impaired patients [11–14]. Subsequently, a recent meta-analysis showed modest improvements in motor scales (HFMSE, RULM and 6MWT) within these populations [15]. However, moderate-to-high heterogeneity across studies suggests variability in clinical practice, patient selection, and evaluators’ training. Furthermore, most studies had a median follow-up of less than three years. Long-term data in adolescent and adults remain scarce and conflicting: while some studies suggest sustained improvement or stabilization [16, 17], others have observed functional deterioration despite nusinersen treatment [18, 19]. Notably, previous research has lacked population-based design and rarely considered treatment withdrawal [16, 20] or included untreated patients as an external comparator [21].

Therefore, this study aims to assess the long-term results of nusinersen treatment specifically addressing the existing data gap in adolescent and adult patients through a population-based approach.

## Methods

### Study design

We conducted a single-center, observational, ambispective, population-based study. The study was performed and reported in accordance with the Strengthening the Reporting of Observational Studies in Epidemiology (STROBE) guidelines for cohort studies [22].

### Population

Currently, all SMA patients living in the Valencian community (ca. 5 million inhabitants) are being treated and followed up at Hospital la Fe. For this study, we retrospectively reviewed all genetically confirmed SMA patients who attended Hospital La Fe, and included all patients living in the Valencian Community between September 2017 (start of the registry) and December 2022. Prevalent cases as of December 2022 were used to calculate point prevalence. Patients were followed up until December 2025 or until death, inclusion in a clinical trial, and switch to another treatment (whichever occurred first). They were excluded from the analysis if they lacked either a pre-treatment evaluation at our center before January 2023, or at least one post-treatment evaluation before the end of follow-up. Demographic, clinical, and genetic data of patients were prospectively collected within CUIDAME National Registry [23] following informed consent (see below). Patients were classified into functional categories: walker (capable to walk without support for 10 m), sitter (able to sit without support for 3 s), or non-sitter. Based on these definitions, patients were assigned their functional status at baseline and at the last observation/censored date.

### Procedures

In Spain, nusinersen has been available since March 2018, with access granted in singular cases under Expanded Access Program (EAP) access from 2017. Risdiplam was first available under compassionate use in 2020 for selected patients with no access to nusinersen (complex spines) and has become widely available under reimbursement since January 2023. Patients were treated according to the Spanish treatment protocol from April 2018 and its posterior revision, based on the Spanish Delphi consensus [24]. This protocol establishes inclusion, exclusion, and discontinuation criteria, together with follow-up recommendations (supplementary material). Nusinersen treatment was indicated by expert neuropediatrician (IPC) in pediatric (< 15 years old) and expert neurologist (JFVC) in adolescent/adult patients, after discussing potential benefits and risks of treatment with the patients (or legal representatives). Nusinersen was delivered as per label by lumbar puncture in 4 loading-dose injections within 2 months and posteriorly every 4 months. Radiological (ultrasound, CT, or fluoroscopy)-guided lumbar puncture was used in patients with complex spines, including spinal fusion, as previously published [25].

Both treated and untreated patients were evaluated every 4–12 months (usually every 8 months), using motor scales according to their age (supplementary material). Although Hammersmith Functional Motor Scale—Expanded (HFMSE) and Revised Upper Limb Module (RULM) were initially developed for children with SMA type 2 and 3, they were later validated in adolescents and adults [26]. However, these scales exhibit floor effects in non-sitters and low-function sitters, as well as ceiling effects in walkers [27]. Ambulatory patients were additionally assessed with the six-minute walk test (6MWT), which has been validated in both children and adult SMA patients [28]. To minimize the floor and ceiling effects of HFMSE and RULM and to enable comparison across different phenotypes, the Spinal Muscular Atrophy Functional Composite Score Revised (SMA-FCR), merging all three scales, was calculated [19]. Adverse events were recorded at each visit.

After two years of treatment (or before if requested by patients) and at every follow-up visit, the continuation of nusinersen was re-evaluated. Clinical benefit was evaluated using both motor and functional scales as previously reported [27]. Patients who showed lack of benefit, as assessed by the clinician and perceived by the patient, or those with an unfavorable risk–benefit balance (considering side effects and patient burden) were proposed to discontinue treatment. All therapeutic decisions were made in agreement with patients. The reasons for discontinuation of treatment were recorded in medical records and retrospectively classified into three categories: (1) lack of perceived benefit (never experienced benefit, as evaluated by clinician and perceived by the patient), (2) loss of an initial benefit, and (3) treatment burden (including adverse events related to the treatment or its administration, but also inconveniences of repeated admissions for lumbar punctures). One single patient could have more than one reason for discontinuation (e.g., loss of benefit and treatment burden).

At the end of follow-up, adolescent and adult patients were classified by the clinical expert using a clinical framework anchored to the 7-point Clinical Global Impression of change (CGI-C) scale and considering both the trajectories of their motor scales and their reports of benefits. According to this, patients were categorized as: sustained responders (exhibiting both sustained patient-reported benefit and a corresponding objective improvement on motor/functional scales), partial responders (reporting a patient-perceived benefit that was not captured on objective scales, or showing a transient initial motor scale improvement that was subsequently lost) and non-responders (those who never reported a perceived benefit or showed any consistent improvement on objective scales).

### Ethics

All patients signed informed consent for participation in CUIDAME registry (*NCT07231549*). This study was approved by the Ethics Committee for Biomedical Research of La Fe Hospital (2019–027-1) in accordance with the Declaration of Helsinki.

### Statistics

Data were summarized as means, standard deviations, medians, and first and third quartiles for the continuous variables, and as relative and absolute frequencies for the categorical variables. Exploratory descriptive analysis and graphs were used to assure the quality of the data and describe the progression of SMA-FCR. Trendlines were estimated using smoothing splines. The first visit was the baseline visit right before starting nusinersen in treated patients or the first overall visit in untreated patients. The last visit was the last available visit before censoring (starting clinical trial or starting a new treatment). For the purpose of this study, patients were divided into 2 subgroups according to age at first visit: < 12 years (children) and ≥ 12 years (adolescents and adults). This age was chosen considering the natural history of the disease (which considerably changes in adolescence) and our experience with nusinersen.

In patients ≥ 12 years, a Bayesian model was developed to assess the independent effect of age, sex, and baseline SMA-FCR in the risk of nusinersen discontinuation. Moreover, a mixed linear regression model was used to compare SMA-FCR trajectories with time in the three subgroups of patients (untreated, treatment discontinuation, and sustained treatment), adjusting for age and sex. In a sensitivity analysis, we also included baseline functional status as a covariate. Statistical analysis and graphs were performed with R software (version 4.5.2).

## Results

**Fig. 1:**
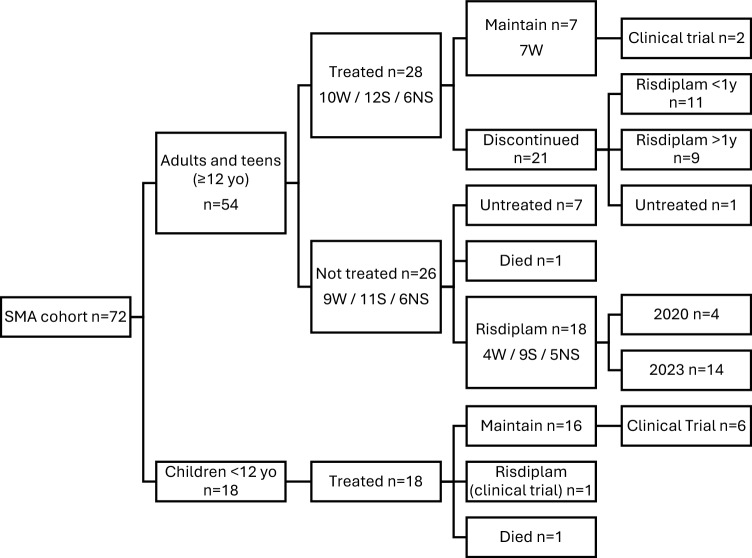
Flow chart of included patients. W – walker, S – sitter, NS – non-sitter; Risdiplam < 1y means switch to risdiplam within one year of nusinersen discontinuation; Risdiplam > 1y means risdiplam started more than 1 year after nusinersen discontinuation

We identified 107 SMA patients visited at Hospital la Fe. 98 of them were living in the Valencian region in December 2022, so the estimated point prevalence was 98 cases/5.059.000 inhabitants = 1,94 cases/100.000 inhabitants. Of the 107 patients, 28 were excluded due to the lack of a baseline visit in our center before January 2023 and 7 because they lacked a follow-up visit. Consequently, 72 patients were finally included in the study and were followed up for a median of 4.6 (IQR 2.7, 5.9) years before censoring. Of those, 18 were < 12 years at the onset of treatment, while 54 were ≥ 12 years (Fig. 1).

By December 2022, 46 SMA patients (64%) had been treated with nusinersen and 26 patients remained untreated. Baseline characteristics of patients can be found in Table 1. Briefly, all children < 12 years were treated (22% with radiological guidance) vs only 28 of 54 (54%) patients ≥ 12 years (46% with radiological guidance). Baseline characteristics of untreated vs. treated patients ≥ 12 years were similar in terms of *SMN2* copies, SMA type, and functional status although untreated patients were older and more frequently female (Table 1). A detailed cross-tabulation of the cohort stratified by age, *SMN2* copy number and treatment can be found in Supplementary Tables S1–S3.

**Table 1:** Baseline characteristics of patients included in the study according to nusinersen treatment

	Nusinersen treated patients			Untreated patients (> 12 yo)*
	All treated	< 12 yo	≥ 12 yo	
*n*	46	18	28	26
Age in years median (IQR)**	14.95 (6.45, 32.08)	3.02 (1.7, 6.9)	27.2 (16.33, 45.25)	35.08 (25.11, 42.85)
Gender F/M n (F%)	26/20 (56%)	14/4 (78%)	12/16 (43%)	17/9 (65%)
SMA type, *n* (%)				
I	2 (4%)	2 (11%)	0	0
II	19 (41%)	9 (50%)	10 (36%)	11 (42%)
III	24 (52%)	6 (33%)	18 (64%)	14 (54%)
IV	0	0	0	1 (4%)
Presymptomatic	1 (2%)	1 (6%)	0	0
SMN2 copies				
1 copy***	1 (2.17%)	0 (0%)	1 (4%)	0 (0%)
2 copies	3 (6.52%)	2 (11.11%)	1 (4%)	0 (0%)
3 copies	31 (67.39%)	14 (77.78%)	17 (61%)	17 (65.38%)
4 copies	11 (23.91%)	2 (11.11%)	9 (32%)	9 (34.62%)
Baseline functional status				
Non-sitter	8 (17.39%)	2 (11.11%)	6 (21.43%)	6 (23.08%)
Sitter	23 (50%)	11 (61%)	12 (42.86%)	11 (42.31%)
Walker	15 (32.61%)	5 (27%)	10 (35.71%)	9 (34.62%)
Baseline SMA-FCR	28.36 (12.22, 53.51), *n* = 38	29.98 (23.94, 51.2), *n* = 10^	28.12 (12.07, 52.37), *n* = 28	19.36 (9.23, 77.7), *n* = 25
Scoliosis	22 (47.83%)	6 (33.33%)	16 (57%)	15 (57.69%)
NIV use	9 (19.57%)	2 (11.11%)	7 (25%)	7 (26.92%)
Radiology assisted lumbar puncture *n*(%)	17 (37%)	4 (22%)	13 (46%)	NA
Follow-up until censoring (months), median (IQR)	55.6 (38.4, 74.98)	51.93 (35.03, 83.86)	56.16 (44.49, 72.75)	49.93 (25.67, 57.6)
Deceased, *n* (age in years)	1 (12)	1 (12)	0	2 (38, 74)

*All untreated patients were older than 12 yo. ** Age at nusinersen's treatment onset for treated or first visit for untreated patients ***SMN1 point mutation ^ Missings (*n* = 8) are due to patients being too young at treatment initiation to be evaluated with SMA-FCR components

The reasons for not starting nusinersen treatment included: lack of intrathecal access despite radiological guidance in four patients (15%), not meeting reimbursement criteria in five individuals (19%) (two lacked residence permit, one had minimal motor function and ≥ 16 h of non-invasive ventilation, and two exhibited normal results in motor scales), and patient’s preference in 17 cases (61%). Of those 26 untreated patients, 18 had started treatment with risdiplam at the end of follow-up, while seven continued untreated and one died due to respiratory insufficiency.

**Fig. 2:**
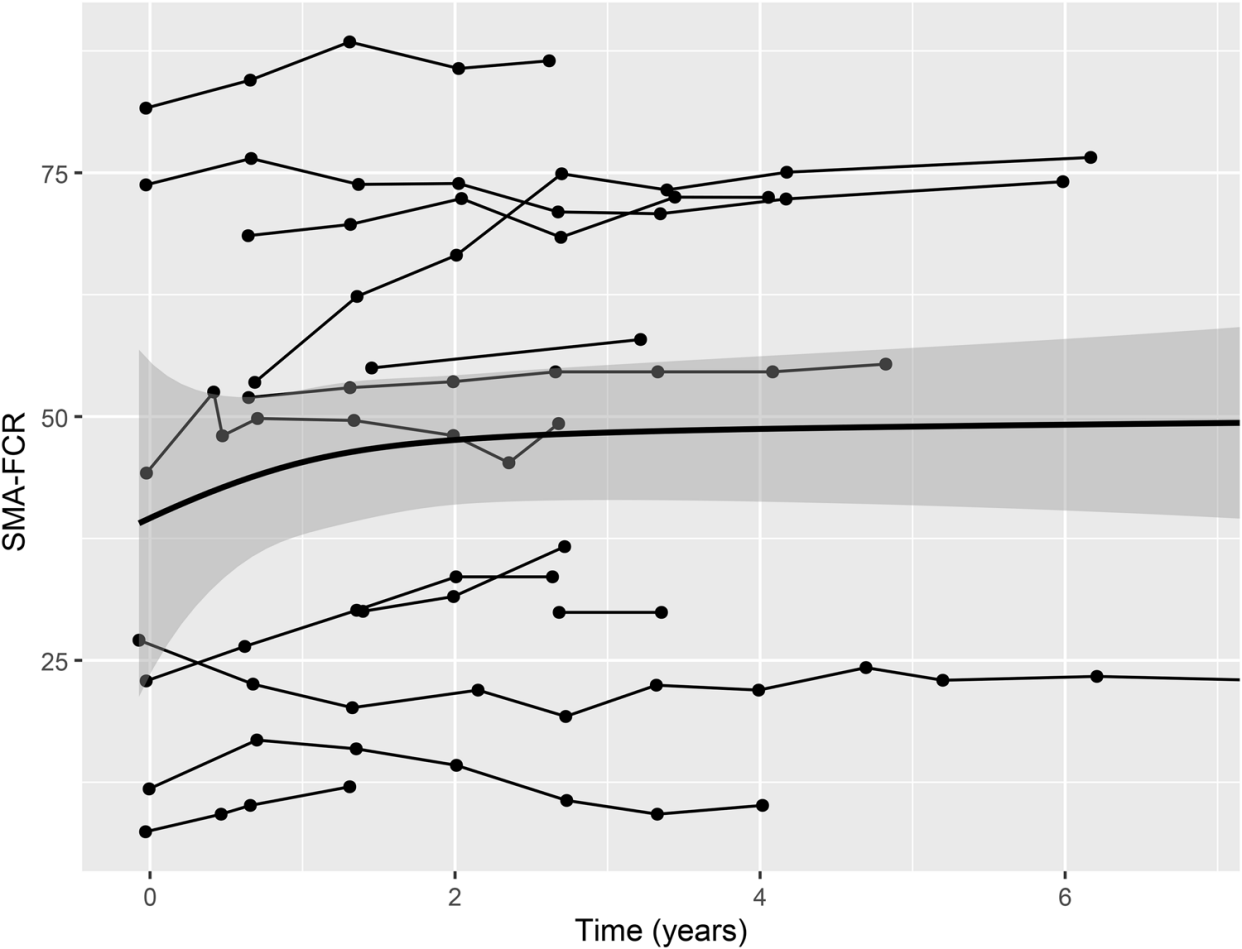
Individual trajectories of SMA-FCR scores in SMA patients: < 12 years old treated with nusinersen; *SMA-FCR* Spinal Muscular Atrophy Functional Composite Score Revised

After a median follow-up of 51.93 months (IQR (35.03, 83.86)), all children < 12 years old were classified as responders at their last visit: three as partial and 15 as sustained responders. Two children discontinued nusinersen treatment during follow-up (Fig. 1): one patient died at the age of 12, after 22 months of therapy due to upper gastrointestinal bleeding, probably unrelated to treatment or SMA; another, discontinued nusinersen to participate in a clinical trial with risdiplam. Additionally, six children treated with nusinersen were included in clinical trials with anti-myostatin molecules and their follow-up data were censored despite continuing on nusinersen. Overall, the SMA-FCR in patients < 12 years showed an improvement during the first two years of treatment followed by stabilization (Fig. 2). In terms of functional transitions, significant milestone gains were observed in this pediatric group: two patients with SMA type 1 achieved independent sitting (transitioning from non-sitter to sitter), and one patient with SMA type 2 achieved independent walking (transitioning from sitter to walker). Furthermore, one presymptomatic patient carrying three SMN2 copies exhibited completely normal development, achieving all motor milestones on time. No pediatric patient experienced a loss of motor milestones during treatment.

In contrast, after a median treatment duration of 56.16 months (IQR 44.49, 72.75) in patients ≥ 12 years old, nine patients (32%) were classified as non-responders, 12 (43%) as partial responders, and seven (25%) as sustained responders (Supplementary Table S2).

Consequently, only seven patients (25%), all of them walkers, remained treated with nusinersen (one of them with radiological guidance, none of them NIV users) at the end of follow-up (Table 2). The remaining 21 patients stopped the treatment after a median of 35 months (IQR 22.4, 52.7). Reasons for discontinuation included: treatment burden reported by 15 patients (71%), loss of benefit in 11 (53%) and/or lack of benefit in nine patients (43%). A detailed cross-tabulation of *SMN2* copy number, baseline functional status, and reasons for discontinuation in patients > 12 years is provided in Supplementary Tables 2 and 4. While non-sitters frequently discontinued due to a mix of non-response and high burden (50.0% each), treatment burden was the most frequent driver for discontinuation among sitters (83.3%) and walkers (100.0%). After discontinuation of nusinersen, 11 patients switched directly to risdiplam after a median time of 52, 73 months (IQR 41.49–66.97). Discontinuations were mostly driven by a combination of treatment burden (9 patients) and loss of benefit (7 patients), while only 3 reported lack of benefit. In contrast, nine patients discontinued treatment before having access to risdiplam treatment. Reasons for discontinuation included treatment burden (6 patients), lack of benefit (5 patients), and loss of benefit (4 patients). One patient stopped treatment due to burden and continues untreated until now.

**Table 2:** Demographic and clinical characteristics of adolescent and adult patients treated with nusinersen

	Nusinersen discontinued (> 12 yo)	Nusinersen maintained (> 12 yo)
N	21	7
Age in years median (IQR)*	29.54 (15.24, 50.17)	22.35 (19.48, 29.58)
Gender F/M n (F%)	11/10 (52%)	1/6 (15%)
Follow-up until censoring in months, median (IQR)	55.73 (40.07, 72.03)	56.9 (46.07, 69.87)
SMN2 copies		
1 copy***	1 (4.76%)	0 (0%)
2 copies	1 (4.76%)	0 (0%)
3 copies	14 (66.67%)	3 (42.86%)
4 copies	5 (23.81%)	4 (57.14%)
Functional status		
Non-sitter	6 (28.57%)	0 (0%)
Sitter	12 (57.14%)	0 (0%)
Walker	3 (14.29%)	7 (100%)
Baseline SMA-FCR score	19.53 (9.51, 33.42), *n* = 21	69.58 (69.14, 71.42), *n* = 6
Scoliosis	13 (61.9%)	3 (42.86%)
NIV use	7 (33.33%)	0 (0%)
Radiological-guided lumbar puncture	12 (57.14%)	1 (14.29%)
Response to nusinersen (*n*)	21	7
No	9 (42.86%)	0 (0%)
Partial	10 (47.62%)	2 (28.57%)
Responder	2 (9.52%)	5 (71.43%)

*SMA-FCR* Spinal Muscular Atrophy Functional Composite Score Revised, *NIV* non-invasive ventilation

^*^age at nusinersen's treatment onset, **SMN1 point mutation

Compared with patients maintaining nusinersen treatment, those who discontinued therapy were older, more frequently female (52% vs. 15%), had lower SMA-FCR and required more frequently radiological lumbar puncture (Table 2). Moreover, all patients discontinuing nusinersen had started treatment either after the age of 40 years old or with a baseline SMA-FCR lower than 50 (Supplementary Fig. 1). This association was confirmed in the multivariable Bayesian model, where older age (expEstimate = 1.028 [1.011, 1.055], prob: 1) independently increased the risk of discontinuation, while male sex (expEstimate = 0.148 [0.005, 2.41], prob: 0.902) and higher SMA-FCR scores (expEstimate = 0.84 [0.718, 0.93], prob: 1) reduced that risk. In a sensitivity analysis, the addition of the baseline motor function did not change the direction of the effects (Supplementary Table 5).

**Fig. 3:**
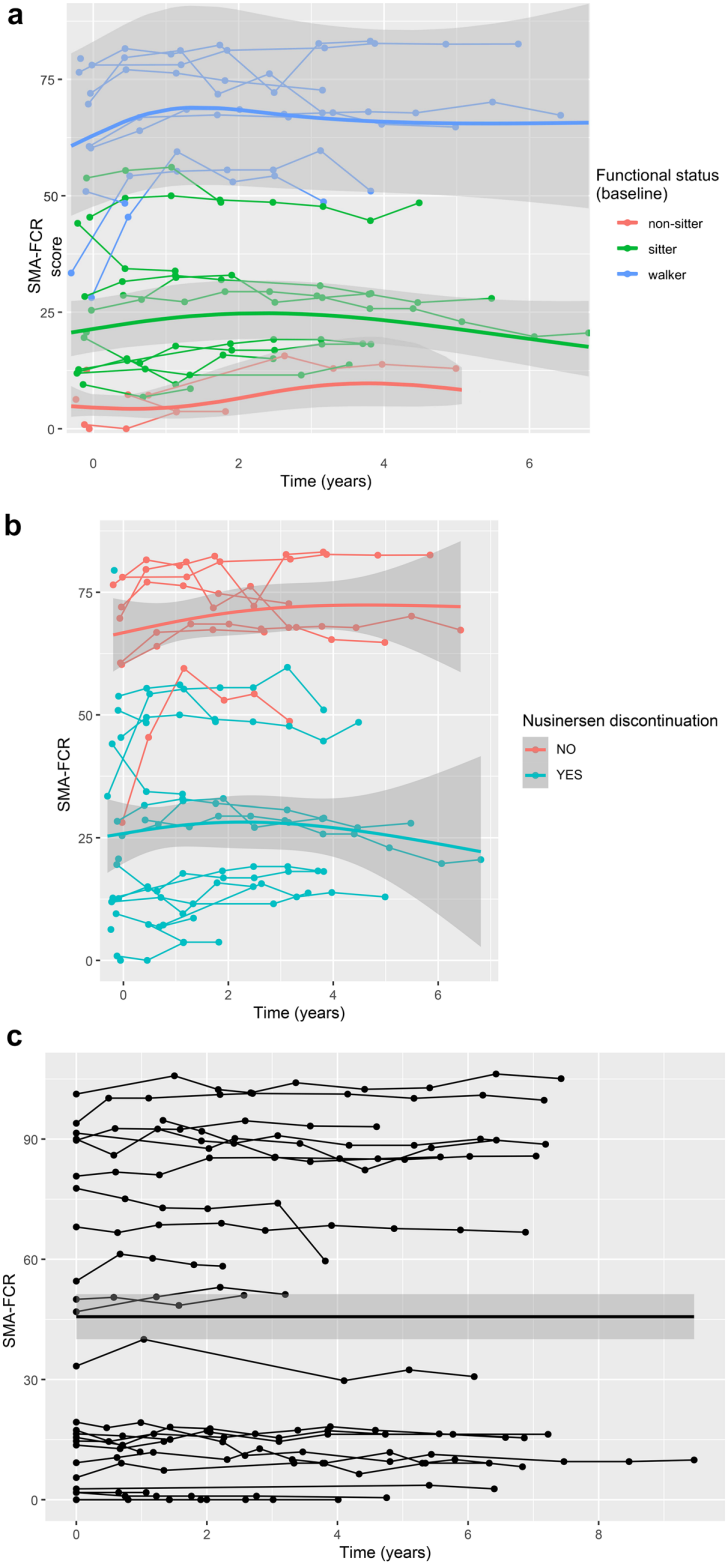
Individual trajectories of SMA-FCR scores in SMA patients ≥ 12 years old: **a** treated with nusinersen according to baseline functional status (b) treated with nusinersen according to its maintenance or withdrawal (**b**) untreated; *SMA-FCR* Spinal Muscular Atrophy Functional Composite Score Revised

**Fig. 4:**
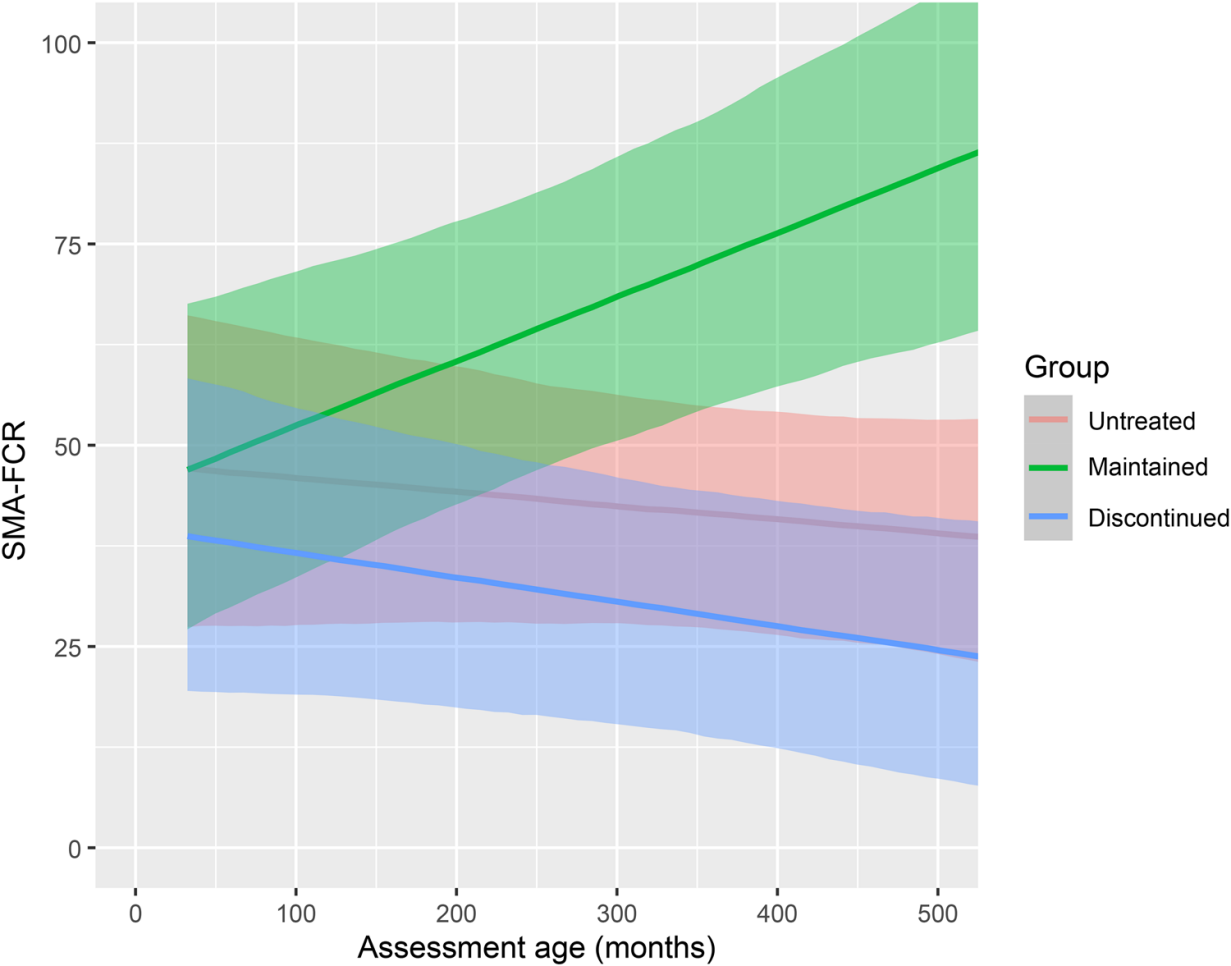
Trajectories of SMA-FCR scores in untreated SMA patients ≥ 12 years compared to those with sustained nusinersen treatment and those who discontinued nusinersen, based on the mixed linear model. *SMA-FCR* Spinal Muscular Atrophy Functional Composite Score Revised

Overall, in walkers ≥ 12 years, an initial increase in SMA-FCR the first 3 years after the start of nusinersen was followed by stability (Fig. 3a). However, in sitters and non-sitters, only stability was observed (Fig. 3a). Remarkably, when stratifying patients by discontinuation, those maintaining nusinersen treatment at last follow-up (*n* = 7) showed a mild increase in SMA-FCR during the first 2 years of treatment followed by stabilization, while those who discontinued treatment (*n* = 21) exhibited stability in SMA-FCR scores (Fig. 3b). Similarly, SMA-FCR remained stable in 26 untreated patients (Fig. 3c). The mixed lineal model (Fig. 4, Supplementary Table S6) confirmed that patients maintaining nusinersen treatment showed a sustained improvement in SMA-FCR scores (+ 0.912 points per year), while patients discontinuing nusinersen showed similar trajectory to those never treated, independently of sex and functional status. Transitions between functional categories were rare among older individuals. In the treated >  = 12 years cohort, a 14-year adolescent non-sitter gained the ability to sit independently after three years of treatment; among adults one patient with SMA type 3, who was a sitter at baseline, regained the capacity to walk after 6 months of therapy but subsequently lost this ability after 4 years of treatment, returning to sitter status. Another treated patient deteriorated from walker to sitter during the course of nusinersen treatment. Within the untreated adult cohort, one patient lost independent walking ability, transitioning from walker to sitter status.

All patients ≥ 12 years old treated with nusinersen presented mild AEs, primarily related to the administration procedure. Additionally, two adult patients developed a moderate AE (neurogenic bladder) during the treatment period: a SMA type 3b male with 4 SMN2 copies, walker who transitioned to sitter, presented with urinary retention. Urodynamic study confirmed detrusor hypocontractility that improved significantly following nusinersen withdrawal. The second patient SMA Type 2b female with 3 SMN2 copies, sitter, who had longer treatment exposure, showed no symptomatic recovery after discontinuation and still requires intermittent catheterization.

## Discussion

This population-based study provides a comprehensive, longitudinal evaluation of nusinersen use, safety, and effectiveness in a real-world setting. We demonstrate that, while overall long-term nusinersen achieves sustained motor improvements in SMA patients, persistence and risk–benefit ratios vary significantly by age and baseline functional status. Within our region, we estimated a prevalence of 1.94 cases per 100.000 inhabitants, which aligns closely with recent estimates from Italy [29] and remains within the established prevalence range for European countries—typically 1–2 cases per 100.000 inhabitants [30]. This suggests that our cohort is highly representative of the broader SMA population, enabling extrapolation of the results to other regions, particularly in Spain.

Population-based studies provide more-balanced results than center-based studies, that restrict inclusion to treated patients and are particularly susceptible to selection bias and confounding by indication [31]. Patients who receive treatment in referral centers often differ systematically from untreated patients in terms of disease severity, comorbidities, or motivation, which can lead to overestimation of treatment effectiveness [32]. This is especially problematic in observational studies, where treatment assignment is non-random and influenced by clinical judgment, patient preferences, or center-specific standards of care practices.

Up till now, population-based studies to assess the effectiveness of nusinersen have been reported only in children. In Sweden, nusinersen was found to increase overall survival in SMA type 1 children [33]. In the Netherlands, an improvement of motor function in 72% and stabilization in another 18% of the symptomatic children treated under 9.5 years old was found [34], which differed from the natural disease course found in a matched retrospective cohort. Our results in children are very similar to that study in terms of use (100% in our study versus 89% in the Dutch study), safety (good tolerance) and effectiveness, with > 90% of responders found in both studies and motor function improving up to two years after treatment onset, followed by stability. This resulted in high persistence of treatment in both studies with few discontinuations, and none of them due to adverse events. The favorable motor outcomes observed in these cohorts also align with the results of open-label and real-world center-based studies [19, 35–39].

However, our findings reveal less favorable outcomes in patients starting nusinersen after 12 years of age. Firstly, only 54% of patients aged ≥ 12 years started treatment with nusinersen between 2018 and 2022, and patient’s preference was the main reason to not initiate treatment. In our experience, the uncertainty about safety and effectiveness in the adulthood made many patients reluctant to receive an intrathecal treatment and many preferred to wait for an oral option. This is in line with studies reporting the preferences of SMA adults for oral treatments compared to intrathecal or intravenous ones [40].

Second, 32% of adolescents and adults were classified as non-responders with 75% discontinuing nusinersen after a median of 34 months. While these figures align with previous US claims analyses [41, 42], they contrast with center-based studies, where discontinuation is infrequently reported or ranges from 0 to 16% with only one study reporting 33% rate [15]. Such discrepancies raise questions about the influence of possible publication or referral bias in center-based studies, shorter follow-up, and differences in the availability of alternative treatments. For example, another center-based study reported 57.7% patients being switched to risdiplam [43].

Notably, 71% of patients in our study reported treatment burden as a primary driver for discontinuation. In many patients, the invasive nature of repeated intrathecal administrations may outweigh the perceived benefit, not only with coexisting severe scoliosis. This is further reflected by 39% of patients who transitioned to risdiplam once it became available. However, the introduction of an oral alternative was only one of the possible reasons as 35% of patients discontinued nusinersen prior to having access to risdiplam. Furthermore, reports of a lack of benefit (43%) and a loss of benefit (53%) suggest that long-term efficacy may evolve over time. Interestingly, the mixed model showed no significant differences in SMA-FCR trajectories between patients discontinuing nusinersen and untreated patients, suggesting that the decision to stop treatment was related with lack or loss of clinical benefit.

While some center-based studies suggested large improvements in adolescents and adults [15, 17, 43–45], long-term open-label clinical trial (SHINE study) [46] and other large observational study [18] have shown that, after 3 years of follow-up, adolescent and adult patients start worsening in motor scales despite nusinersen treatment. It is often argued that, in the context of a neurodegenerative condition, long-term stability or deceleration of decline is a therapeutic response. While we overall agree with this statement, our study shows that extended periods of stability in motor scales also occurred within our untreated patients group. This observation suggests that long *stability* periods may be part of the natural history of the disease in certain adult patients.

On the other hand, we observed an improvement in SMA-FCR scores followed by a sustained stability in 25% of adolescents and adults, which is unlikely in the natural history of the disease. The mixed model analysis further confirmed a different SMA-FCR trajectory in those patients compared to untreated patients. This suggests that nusinersen remains a good option for a subset of adolescent and adult patients; identifying these individuals is essential to optimizing the risk–benefit ratio of the treatment.

In our cohort, adolescents and adults who maintained long-term treatment were predominantly male, younger than 40 years, ambulatory, and presented with mild scoliosis, and a robust initial response. The independent effect of age, male sex, and higher motor function in treatment discontinuation was confirmed in a multivariable analysis. Higher baseline motor function and ambulatory status have been previously associated with superior outcomes in adults [12, 13, 18, 21], suggesting that a greater functional reserve of remaining motor units, is a prerequisite for translating nusinersen mechanism of action into clinically meaningful improvements.

However, our finding that ambulatory status and higher baseline function strongly dictate long-term persistence is more clear-cut and categorical than in previous large multicenter cohorts [45]. This difference is likely driven by our significantly extended follow-up period (median 4.6 years vs. 1–2 effective years in earlier studies), allowing long-term trends to fully manifest as discontinuations. Furthermore, the population-based design avoids selection bias and the single-center management of our population ensured a more uniform application of discontinuation criteria, contrasting with the heterogeneous clinical thresholds inherent to multicenter registries.

Long-term treatment maintenance was more frequent among patients with higher SMN2 copy number. However, our data suggest that long-term persistence and efficacy are driven primarily by the remaining structural motoneuron reserve rather than copy number, as illustrated by the adult non-sitter in our cohort who carried 4 SMN2 copies but was a non-responder and discontinued therapy, or the two pediatric patients carrying 2 SMN2 copies, who were responders. Thus, age and baseline functional status (as proxies of motoneuron reserve) at treatment initiation remain the primary drivers of long-term persistence to nusinersen therapy.

Although age at treatment initiation is critical in children, its independent influence in older patients remains debated [12, 13, 18, 21]. Our study suggests an independent effect on discontinuations, but this might be driven by a higher treatment burden in older patients. Interestingly, sex has not been previously linked to treatment efficacy; the association of age and female sex with nusinersen discontinuation found in our study probably reflects the greater burden of lumbar punctures with aging and the fact that female sex is a well-documented risk factor for post-lumbar puncture headache [47].

Importantly, we report the development of moderate neurogenic bladder dysfunction in two of our treated adult patients. While bladder and voiding disturbances, particularly correlating with more severe phenotypes, are likely underrecognized in SMA cohorts [48, 49], repeated lumbar puncture may also lead to development or exacerbation of these symptoms. The clinical trajectories of our two patients suggest a possible treatment-related adverse event, but an overlap with an underlying systemic SMA manifestation is also plausible.

In the absence of reliable predictive or response biomarkers in adolescent and adults [50], variables, such as age, sex, and baseline motor function, should be considered for personalizing therapeutic decision and managing patient expectations [51]. Our findings reinforce the growing body of evidence that a significant proportion of SMA patients face unmet needs, even with the availability of disease-modifying therapies [52]. This highlights the need for exploring new therapeutic interventions. Since most patients who discontinued nusinersen pointed to treatment burden as the main factor, considering alternatives like oral treatments, intrathecal port devices (*NCT06555419*) or less frequent, annual intrathecal dosing (*NCT07221669*) could enhance persistence. Moreover, new options should also be explored to reduce the high economic burden of drugs for patients who show little functional gain from therapy.

### Strengths and limitations

Limitations include the single-center nature of the follow-up, which might introduce specialist preference bias, though the motor scale results suggest this bias was not relevant. Additionally, while the sample size allowed for multivariable analysis, comparisons between subgroups should be interpreted with caution. Finally, the evaluation of treatment efficacy remains a challenge, particularly in adults and low-function patients [5]. The use of SMA-FCR in this study has allowed for a better comparison of broad cohorts, but still only considers motor function, disregarding bulbar, respiratory, or fatigue evaluation, and does not reflect patient perspective [27, 53]. Thus, SMA-FCR might be insufficient to capture the complexity of possible treatment’s benefits.

However, the validity and reliability of the SMA-FCR scale in capturing this functional continuum is strongly supported by recent literature. Notably, our cohort’s functional data correspond with the median SMA-FCR values reported by Pasternak et al. (2025), confirming the external consistency of this composite metric in real-world cohorts.

Moreover, the Minimal Clinically Important Difference (MCID) threshold was not applied for the definition of responder as meaningful clinical thresholds vary substantially depending on baseline age and functional status [54] and, for severely impaired patients, even minor changes not captured by motor scales can be highly significant.

A major strength is the population-based design, which limits the potential selection bias toward more motivated and proactive individuals. By including data on patients who withdrew from or never initiated treatment, we provide a more realistic reflection of nusinersen’s real-world impact than studies that focus only on those who remain on therapy.

## Conclusion

In this long-term, population-based study, nusinersen persistence was high in children but substantially low after the age of 12. While treatment discontinuation was primarily driven by the high procedural burden and limited perceived effectiveness, the subset of patients who maintained nusinersen treatment showed sustained benefit. Among adolescents and adults, younger age and higher baseline function were key predictors of a more favorable outcome. Integrating these factors into clinical decision-making will help to personalize therapeutic strategies and set realistic expectations for patients and families. Future research should involve extended longitudinal observations and a broad range of outcome measures, with a specific focus on identifying predictive and response biomarkers to optimize care across the entire SMA spectrum.

## Supplementary Information

Below is the link to the electronic supplementary material.

Supplementary file1 (DOCX 109 KB)

## Data Availability

The data that support the findings of this study are available from the corresponding author upon reasonable request.
