# Supplementary material for: Long-term persistence, safety and effectiveness of nusinersen in spinal muscular atrophy: a population-based study
Source: J Neurol. 2026 Jun 29;273(7):430. doi: 10.1007/s00415-026-13965-0 (PMC13315468; doi:10.1007/s00415-026-13965-0)

Supplementary Material

Methodology

Spanish SMA treatment protocol criteria

In pediatric patients, inclusion criteria included all symptomatic patients and those presymptomatic with 0-3 copies. In adult patients, inclusion criteria included symptomatic patients with an objective impairment in the evaluation or scales and evidence of disease progression in the last 5 years (as per patient report or medical reports). Exclusion criteria were off-label use (including patients without lumbar access), patients with very severe impairment (minimal function and ≥16 hours of non-invasive ventilation -NIV-) and unfavourable risk-benefit ratio as per clinical judgement. In our region, patients not meeting criteria could also receive treatment, if clinically justified, on a case-by-case basis and after local approval.

Motor scales

CHOPINTEND, Hammersmith Functional Motor Scale - Expanded (HFMSE) and Revised Upper Limb Module (RULM) and 6-Minute Walk Test (6MWT) were used to evaluate patients at the appropriate age.

The HFMSE, which consists of 33 items, with a maximum of 66 points (higher scores indicating better function). This scale was originally designed for the assessment of high- functioning type 2 and 3 SMA patients, that is, sitters and walkers, although it shows floor effect in low-function sitters [31]. The RULM, which includes 20 items with a maximum score of 37 (higher scores indicating better function). Although it has been validated in both ambulant and non- ambulant patients, it shows a ceiling effect in up to a third of ambulant patients with SMA type 3 and a floor effect in a proportion of non- sitters [31].

Results:

**Descriptive tables by age of treatment onset**

**Table S1. Nusinersen age at onset <12 years**

*Values are presented as n (% within each SMN2 copy-number column) for categorical variables and as summary statistics for age at onset. Group size: n=18.*

| **Variable** | **Category** | **SMN2 = 2 (n=2)** | **SMN2 = 3 (n=14)** | **SMN2 = 4 (n=2)** |
| --- | --- | --- | --- | --- |
| **Female Sex** | Yes | 2 (100.0%) | 11 (78.6%) | 1 (50.0%) |
| **SMA type** | 1b | 2 (100.0%) | 0 (0.0%) | 0 (0.0%) |
|  | 2a | 0 (0.0%) | 5 (35.7%) | 0 (0.0%) |
|  | 2b | 0 (0.0%) | 4 (28.6%) | 0 (0.0%) |
|  | 3a | 0 (0.0%) | 4 (28.6%) | 2 (100.0%) |
|  | Presymptomatic | 0 (0.0%) | 1 (7.1%) | 0 (0.0%) |
| **Functional status (pre)** | Non-sitter | 2 (100.0%) | 0 (0.0%) | 0 (0.0%) |
|  | Sitter | 0 (0.0%) | 11 (78.6%) | 0 (0.0%) |
|  | Walker | 0 (0.0%) | 3 (21.4%) | 2 (100.0%) |
| **Functional status (post)** | Non-sitter | 0 (0.0%) | 0 (0.0%) | 0 (0.0%) |
|  | Sitter | 2 (100.0%) | 10 (71.4%) | 0 (0.0%) |
|  | Walker | 0 (0.0%) | 4 (28.6%) | 2 (100.0%) |
| **Response to nusinersen** | Responder | 1 (50.0%) | 12 (85.7%) | 2 (100.0%) |
|  | Partial responder | 1 (50.0%) | 2 (14.3%) | 0 (0.0%) |
| **Nusinersen discontinued** | Yes | 0 (0.0%) | 2 (14.3%)* | 0 (0.0%) |

*Abbreviations: SMA, spinal muscular atrophy; IQR, interquartile range; SD, standard deviation. *One patient discontinued to participate in a clinical trial (MANATEE), another patient died while on nusinersen.*

**Table S2. Nusinersen age at onset** ≥ **12 years**

*Values are presented as n (% within each SMN2 copy-number column) for categorical variables and as summary statistics for age at onset. Group size: n=28.*

| **Variable** | **Category** | **SMN2 = 1 (n=1)** | **SMN2 = 2 (n=1)** | **SMN2 = 3 (n=17)** | **SMN2 = 4 (n=9)** |
| --- | --- | --- | --- | --- | --- |
| **Female Sex** | Yes | 1 (100.0%) | 0 (0.0%) | 9 (52.9%) | 2 (22.2%) |
| **SMA type** | 2a | 0 (0.0%) | 0 (0.0%) | 6 (35.3%) | 0 (0.0%) |
|  | 2b | 1 (100.0%) | 0 (0.0%) | 3 (17.6%) | 0 (0.0%) |
|  | 3a | 0 (0.0%) | 0 (0.0%) | 6 (35.3%) | 1 (11.1%) |
|  | 3b | 0 (0.0%) | 1 (100.0%) | 2 (11.8%) | 8 (88.9%) |
| **Functional status (pre)** | Non-sitter | 1 (100.0%) | 0 (0.0%) | 4 (23.5%) | 1 (11.1%) |
|  | Sitter | 0 (0.0%) | 1 (100.0%) | 9 (52.9%) | 2 (22.2%) |
|  | Walker | 0 (0.0%) | 0 (0.0%) | 4 (23.5%) | 6 (66.7%) |
| **Functional status (post)** | Non-sitter | 1 (100.0%) | 0 (0.0%) | 3 (17.6%) | 1 (11.1%) |
|  | Sitter | 0 (0.0%) | 1 (100.0%) | 10 (58.8%) | 3 (33.3%) |
|  | Walker | 0 (0.0%) | 0 (0.0%) | 4 (23.5%) | 5 (55.6%) |
| **Response to nusinersen** | Responder | 0 (0.0%) | 0 (0.0%) | 4 (23.5%) | 3 (33.3%) |
|  | Partial responder | 0 (0.0%) | 0 (0.0%) | 8 (47.1%) | 4 (44.4%) |
|  | No | 1 (100.0%) | 1 (100.0%) | 5 (29.4%) | 2 (22.2%) |
| **Nusinersen discontinued** | Yes | 1 (100.0%) | 1 (100.0%) | 14 (82.4%) | 5 (55.6%) |
| **Reasons for discontinuation** | Lack of benefit | 1 (100.0%) | 1 (100.0%) | 5 (29.4%) | 2 (22.2%) |
|  | Loss of benefit | 0 (0.0%) | 0 (0.0%) | 8 (47.1%) | 3 (33.3%) |
|  | Nusinersen burden | 1 (100.0%) | 0 (0.0%) | 11 (64.7%) | 4 (44.4%) |

*Abbreviations: SMA, spinal muscular atrophy; IQR, interquartile range; SD, standard deviation.*

**Table S3. Untreated patients**

*Values are presented as n (% within each SMN2 copy-number column) for categorical variables and as summary statistics for age at onset. Group size: n=26.*

| **Variable** | **Category** | **SMN2 = 3 (n=17)** | **SMN2 = 4 (n=9)** |
| --- | --- | --- | --- |
| **Female Sex** | Yes | 12 (70.6%) | 5 (55.6%) |
| **SMA type** | 2a | 5 (29.4%) | 0 (0.0%) |
|  | 2b | 6 (35.3%) | 0 (0.0%) |
|  | 3a | 4 (23.5%) | 2 (22.2%) |
|  | 3b | 2 (11.8%) | 6 (66.7%) |
|  | 4 | 0 (0.0%) | 1 (11.1%) |
| **Functional status (pre)** | Non-sitter | 6 (35.3%) | 0 (0.0%) |
|  | Sitter | 8 (47.1%) | 2 (22.2%) |
|  | Walker | 3 (17.6%) | 7 (77.8%) |
| **Functional status (post)** | Non-sitter | 6 (35.3%) | 0 (0.0%) |
|  | Sitter | 9 (52.9%) | 2 (22.2%) |
|  | Walker | 2 (11.8%) | 7 (77.8%) |

*Abbreviations: SMA, spinal muscular atrophy; IQR, interquartile range; SD, standard deviation.*

Table S4. Detailed responder-status and reasons for discontinuation.

| Baseline Functional Status | Discontinuation rate | Non-Responders | Partial Responders | Responders | Lack of Benefit | Loss of Benefit | Treatment Burden |
| --- | --- | --- | --- | --- | --- | --- | --- |
| Non-Sitters | 6/6 (100%) | 3 (50.0%) | 2 (33.3%) | 1 (16.7%) | 3 (50.0%) | 3 (50.0%) | 3 (50.0%) |
| Sitters | 12/12 (100%) | 4 (33.3%) | 7 (58.3%) | 1 (8.3%) | 4 (33.3%) | 7 (58.3%) | 10 (83.3%) |
| Walkers | 3/10 (30%) | 2 (66.7%) | 1 (33.3%) | 0 (0.0%) | 2 (66.7%) | 1 (33.3%) | 3 (100.0%) |
| Total | 21/28 (75%) | 9 (42.9%) | 10 (47.6%) | 2 (9.5%) | 9 (42.9%) | 11 (52.4%) | 16 (76.2%) |

Table S5. Bayesian model assessing the independent effect on the risk of nusinersen discontinuation in patients ≥12 years old adjusting by age, sex, baseline SMA-FCR and baseline functional status

| **Variables** | **exp.Estimate.** | **Lower.95.** | **Upper.95.** | **pd** |
| --- | --- | --- | --- | --- |
| Age | 1.027 | 1.01 | 1.058 | 1 |
| Male sex | 0.787 | 0.109 | 2.634 | 0.608 |
| SMA-FCR | 0.886 | 0.771 | 0.998 | 0.978 |
| Sitter | 1.426 | 0.344 | 18.497 | 0.629 |
| Walker | 0.043 | 0 | 1.386 | 0.836 |

Table S6. Multivariable mixed beta model assessing the trajectories of SMA-FCR with time, according to treatment subgroups (maintained, discontinued, untreated) and adjusting by sex and functional status. In bold, statistically significant results.

| **Variables** | **Estimate** | **Std..Error** | **Lower.95.** | **Upper.95.** | **pd** |
| --- | --- | --- | --- | --- | --- |
| Male sex | 0.087 | 2.834 | -5.378 | 5.741 | 0.505 |
| Time (months) | -0.012 | 0.015 | -0.041 | 0.015 | 0.791 |
| Maintained | 0.054 | 2.902 | -5.541 | 5.684 | 0.509 |
| Discontinuation | -0.294 | 2.865 | -5.85 | 5.341 | 0.543 |
| e_funcionalsitter | -2.052 | 2.81 | -7.735 | 3.432 | 0.764 |
| **e_funcionalwalker** | **5.866** | **3.002** | **0.083** | **11.791** | **0.976** |
| **Time:matained** | **0.088** | **0.023** | **0.043** | **0.135** | **1** |
| Time:discontinued | -0.022 | 0.018 | -0.057 | 0.013 | 0.886 |

Supplementary Figure 1

Individual SMA-FCR trajectories of patients sustaining and discontinuing nusinersen according to their age at baseline


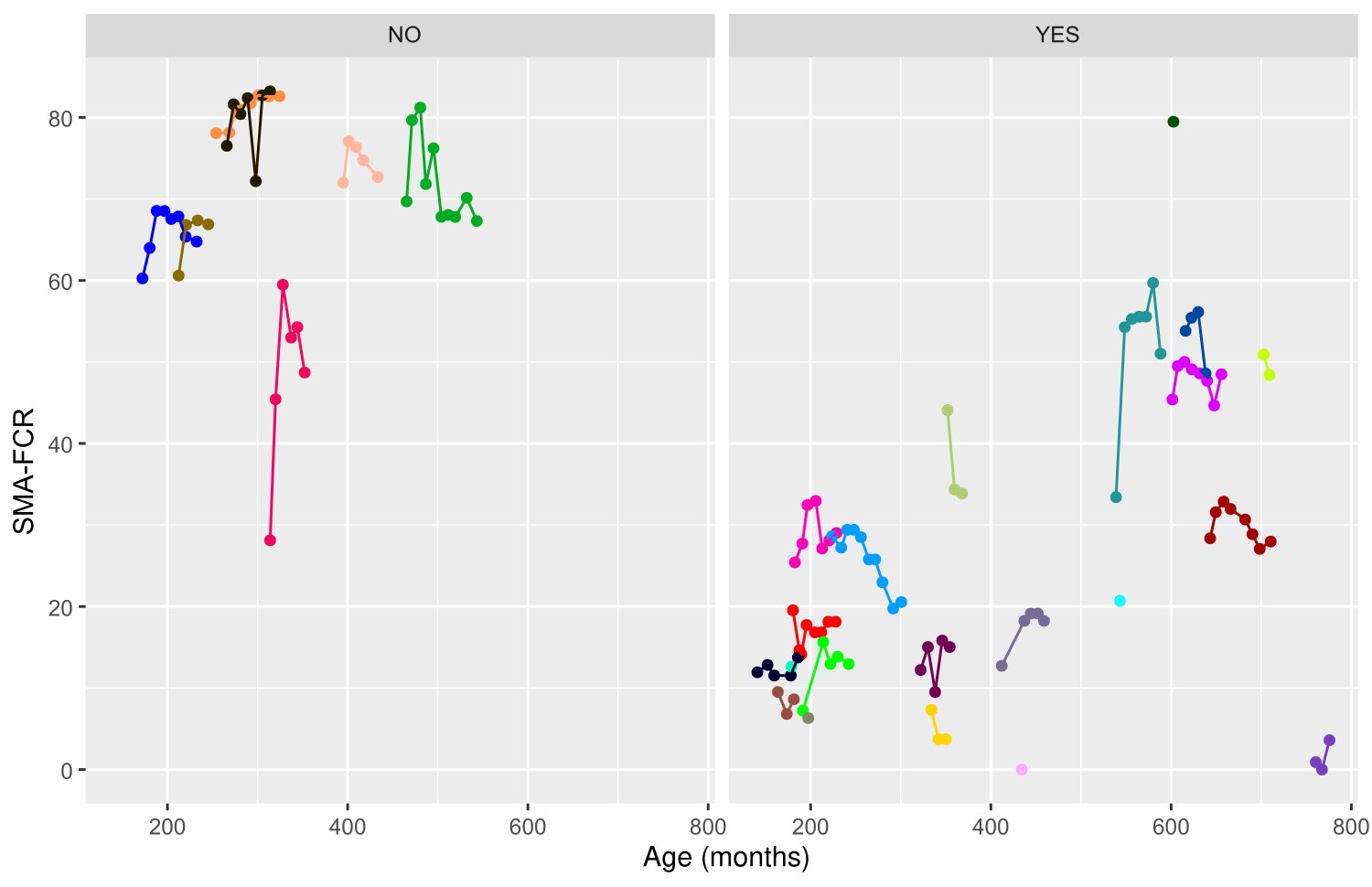

Supplement: Supplementary file 1 — Supplementary file1 (DOCX 109 KB) [file 415_2026_13965_MOESM1_ESM.docx]
